# Single cell RNA-sequencing data generated from mouse adipose tissue during the development of obesity

**DOI:** 10.1016/j.dib.2024.110119

**Published:** 2024-02-01

**Authors:** Zhimin Lu, Ling Ding, Xuewen Tian, Qinglu Wang

**Affiliations:** College of Sport and Health, Shandong Sport University, Jinan, Shandong 250102, China

**Keywords:** Obesity development, Adipose tissue, Immune microenvironment, Inflammation

## Abstract

In recent years, the number of obesity has increased rapidly around the world, and it has become a major public health problem endangering global health [1]. Obesity is caused by excessive calorie intake over a long period of time, and high-fat diet (HFD) is one of the important predisposing factors [2], [3], [4]. Adipose tissue (AT) is an important immune and endocrine organ in the body, and plays an important role in the body [5]. Obesity leads to AT dysfunction, AT dilation and cell hypertrophy. Dysfunctional fat cells are the main source of pro-inflammatory cytokines, which aggravate low-grade systemic inflammation and further promote the development of obesity-related diseases [6], [7], [8]. However, whether AT releases pro-inflammatory cytokines in the early stages of obesity development remains unknown. The AT microenvironment is composed of a variety of cells, including fat cells, immune cells, fibroblasts, and endothelial cells. The immune microenvironment (TIME) and its metabolic imbalance can lead to the secretion or regulation of related hormones, which causes inflammation AT [9]. TIME is very important for maintaining AT homeostasis, which is crucial for the occurrence of obesity [10,11]. This data use single-cell RNA sequencing (sNuc-Seq) to analyze the characteristics of TIME changes in the mouse epididymal adipose tissue during the development of obesity, and the changes of cell types and genes in the tissue.

Specifications Table

**Table:** 

Subject	Biology
Specific subject area	Single nucleus transcriptome sequencing, Molecular Biology, Cell Biology, mouse epididymal adipose tissue.
Data format	Raw, controlled transcriptome sequencing data
Type of data	Table, Image, Chart, Graph, Figure
Data collection	The 10x Genomics platform uses microfluidic technology to enclose the bead and cells with Cell Barcode in the droplet, collect the droplet containing cells, and then split the cells in the droplet, so that the mRNA in the Cell is connected with the Cell Barcode above the bead. Single Cell GEMs are formed, reverse transcription reaction is performed in the droplet, cDNA library is constructed, and sample source of target sequence is distinguished by sample index on the library sequence.
Data source location	Shandong Sport University, Jinan, Shangdong, China
Data accessibility	Repository name: GEO NCBI Data identification number: GSE235469 Direct URL to data: https://www.ncbi.nlm.nih.gov/geo/query/acc.cgi?acc=GSE235469

## 1 Value of the Data

• These single cell RNA-sequencing profiles, obtained from the epididymal adipose tissue during the development of mouse obesity, which can explain the cell types and gene changes in the epididymal adipose tissue during the development of mouse obesity.
• The exploration of these data will provide molecular insights into the changes in adipose tissue microenvironment triggered by obesity, clarify the stage of adipose tissue inflammation and the mechanism of adipose tissue neuron apoptosis, which will be useful for researchers studying the occurrence and development of adipose tissue inflammation caused by obesity.
• These data can be further analyzed to better understand the regulatory mechanism of “fibroblast-neutrophil-macrophage-neuron” crosstalk in adipose tissue and guide the treatment of obesity and complications.

## 2 Objective

The Objective of this study was to analyze the internal mechanism of adipose tissue changes during obesity formation by single cell sequencing, to provide theoretical support for the treatment of obesity and its complications, and to provide help for relevant researchers.

## 3 Data Description

**Fig. 1:**
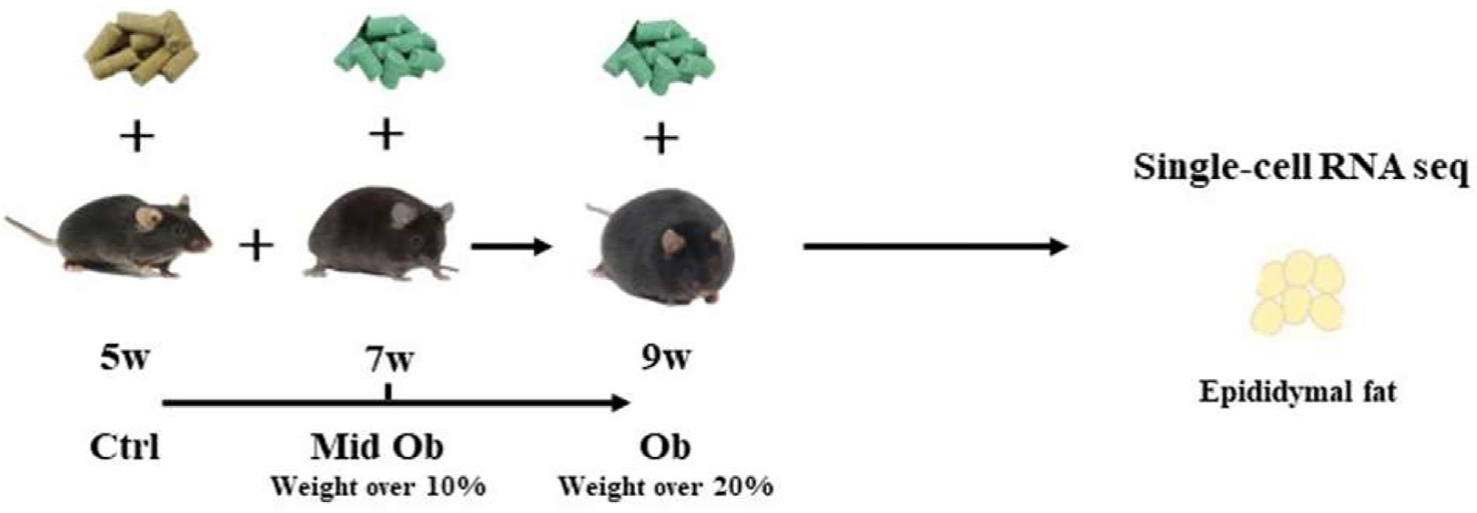
Establishment of obese mouse model.

This dataset contains data of mouse epididymal adipose tissue during the development of obesity, including data at three stages: before obesity (Ctrl group), during obesity development (Mid_Ob group), and during obesity formation (Ob group) (Fig. 1). Table 1 described the data storage location. Table 2 shows the genome sequencing data of mouse epididymal adipose tissue. This analysis completed single-cell transcriptome sequencing of 3 samples, and the number of high-quality Cell Ranger cells in each sample was distributed in the range of 5021–14,621 for quantitative quality control. After quality control such as double cell, multicellular and apoptotic cells were eliminated, Finally, the number of cells obtained was distributed in the range of 4442–11,672, the average UMI number in each cell was distributed in the range of 6230–10,619, the average gene number in each cell was distributed in the range of 2480–3449, the average mitochondrial UMI ratio in each cell was distributed in the range of 0.0030–0.0050. Table 3 shows the list of accession number of epididymal adipose tissue in mouse in GEO database.

**Table 1:** Data storage location.

Item	Raw file	Processed data file
Ctrl	OES216806150B-01_S1_L001_R1_001.fastq OES216806150B-01_S1_L001_R2_001.fastq	Ctrl differential_expression.xlsx
Mid_Ob	OES217064150B-01_S1_L001_R1_001.fastq OES217064150B-01_S1_L001_R2_001.fastq	Mid_Ob differential_expression.xlsx
Ob	OES216807150B-01_S1_L001_R1_001.fastq OES216807150B-01_S1_L001_R2_001.fastq	Ob differential_expression.xlsx

**Table 2:** Single nucleus transcriptome sequencing results of epididymal adipose tissue of 3 groups.

Item	Value
Coverage (×)	10 ×
High-quality cell number distribution after quality control	5021–14,621
The final number of cells obtained	4442–11,672
The average UMI in each cell	6230–10,619
The average number of genes in each cell	2480–3449
Average mitochondrial UMI distribution in each cell	0.030–0.050

**Table 3:** List of accession number of epididymal adipose tissue in mouse in GEO database.

Sample	Type	Treatment	Geo accession number
Ctrl	Adipose tissue	General diet	GSM7502687
Mid_Ob	Adipose tissue	High fat diet	GSM7502688
Ob	Adipose tissue	High fat diet	GSM7502689

## 3 Experimental Design, Materials and Methods

All animal experiments were approved by the Animal Ethics Committee of Shandong Physical Education University. The 5-week-old C57BL/6J male mice were purchased from Jiangsu Huachennuo Medical Technology Co., LTD., and domesticated for 1 week in SPF facilities. The mice were maintained in a temperature-controlled (25°C) facility with a 12-h light/dark cycle. Mice in the control group were fed ordinary diet, and mice in the high-fat diet group were fed 60% high-fat diet. The mice were weighed at a fixed time each week. Normal mice (Ctrl group), during obesity (Mid_Ob group) and after obesity (Ob group) were selected, 8 mice in each group. The criterion for judging the success of Mid_Ob group modeling is that its body weight exceeds 10% of the average body weight of the control group, and the criterion for judging the success of Ob modeling is that its body weight exceeds 20% of the average body weight of the control group [12]. Serum total cholesterol (TC), triglyceride (TG), low density lipoprotein (LDL-C) and high density lipoprotein (HDL-C) C57BL/6J male mice were detected by Elisa method every week. The experimental animals were prohibited from drinking water 12 h before sampling. The mice were injected with peritoneal anesthesia with 3% concentration of pentobarbital sodium according to 1 ml/kg of body weight, and the neck was removed after taking blood from orbit. The abdominal cavity of the mice was opened, the liver was removed, and the epididymal fat was removed with tweezers. Adipose tissue was removed and blood stains were rinsed with 0.9% saline. Eight pieces of subcutaneous adipose tissue and periepididymal adipose tissue (about 300 mg each) were selected from each group and put into a fixed bottle containing 4% paraformaldehyde for histological fixation. The remaining adipose tissue was cut and divided into a labeled 2 ml cryopreservation tube (Thermo, external rotating cryopreservation tube 375418, USA), and immediately frozen in liquid nitrogen. After sampling, the samples were transferred to the −80 °C refrigerator for single-cell sequencing. We mixed the parepididymal adipose tissue of 8 mice from each group into a tube for single-cell sequencing to avoid some errors caused by the experimental process.

**Fig. 2:**
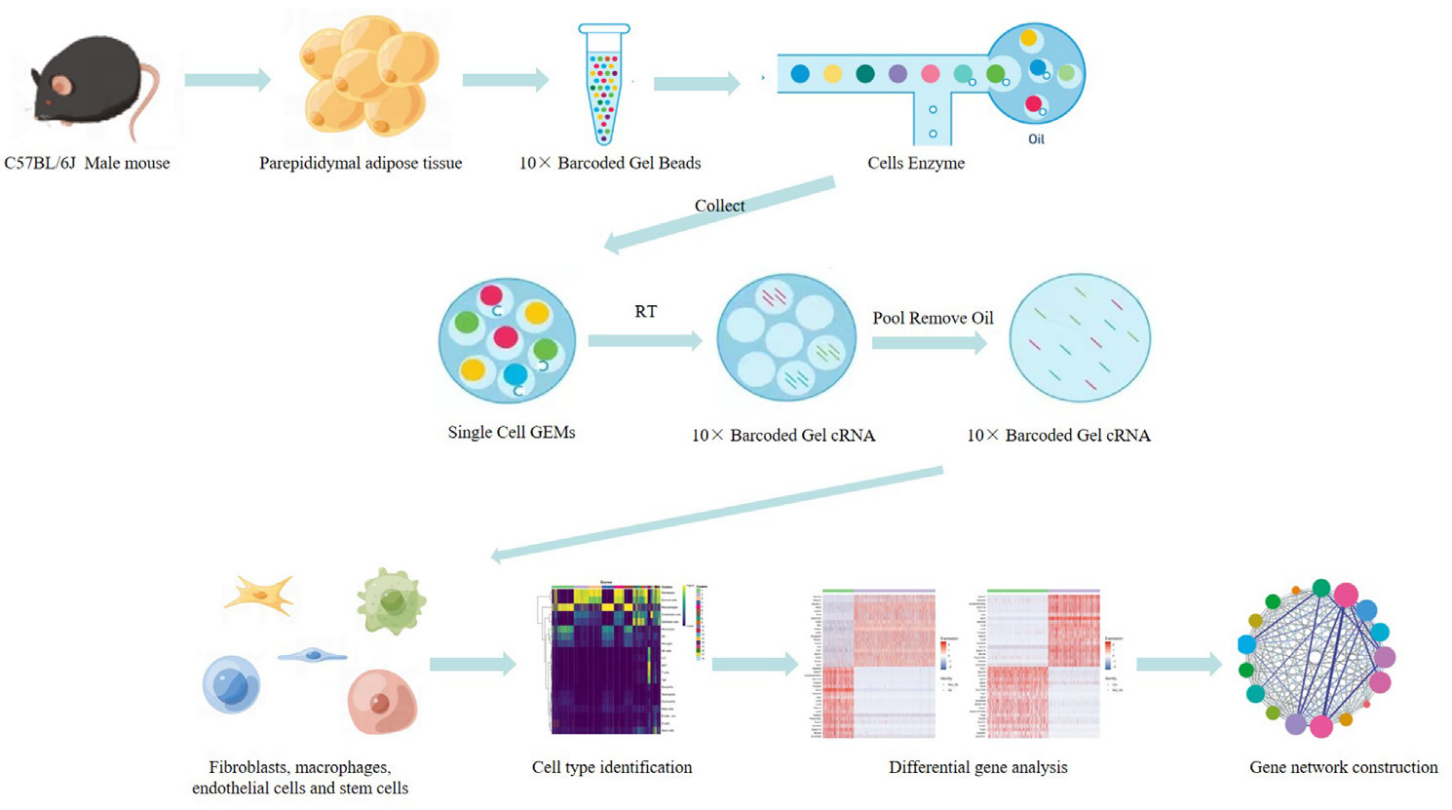
Schematic diagram of the main process of data acquisition.

**Fig. 3:**
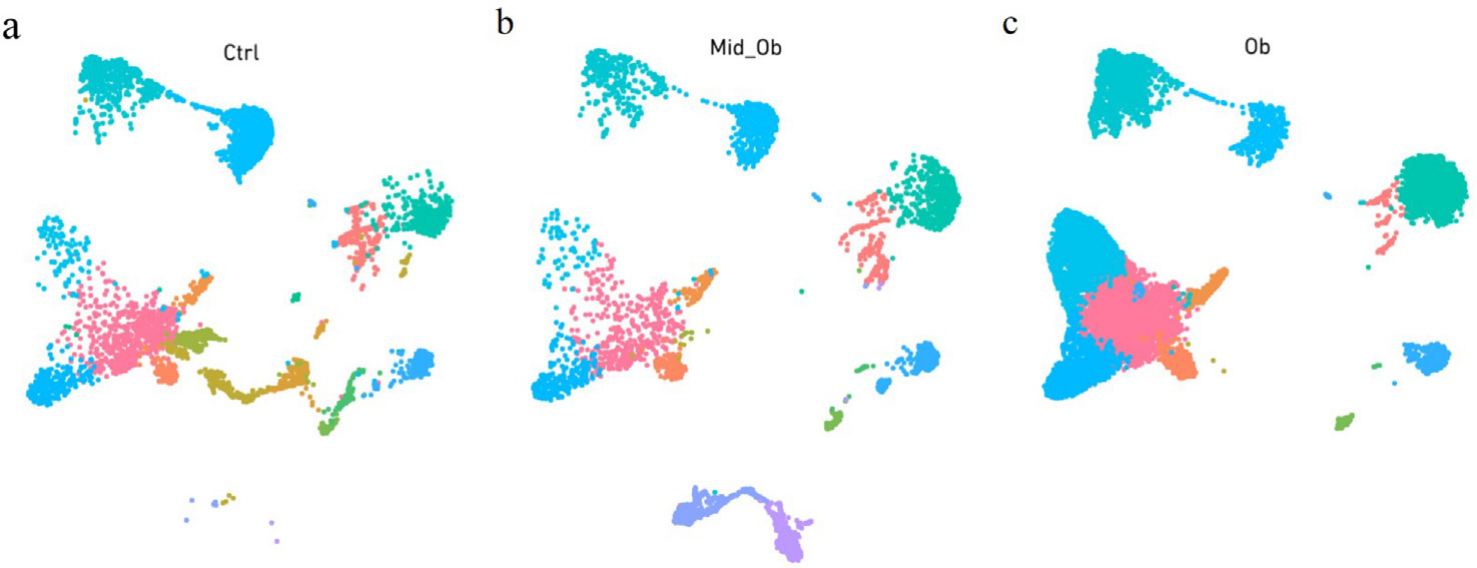
Adipose tissue is divided into 18 subgroups. a. Dataset GSM7502687 adipose tissue. b. Dataset GSM7502688 adipose tissue. c. Dataset GSM7502689 adipose tissue.

**Fig. 4:**
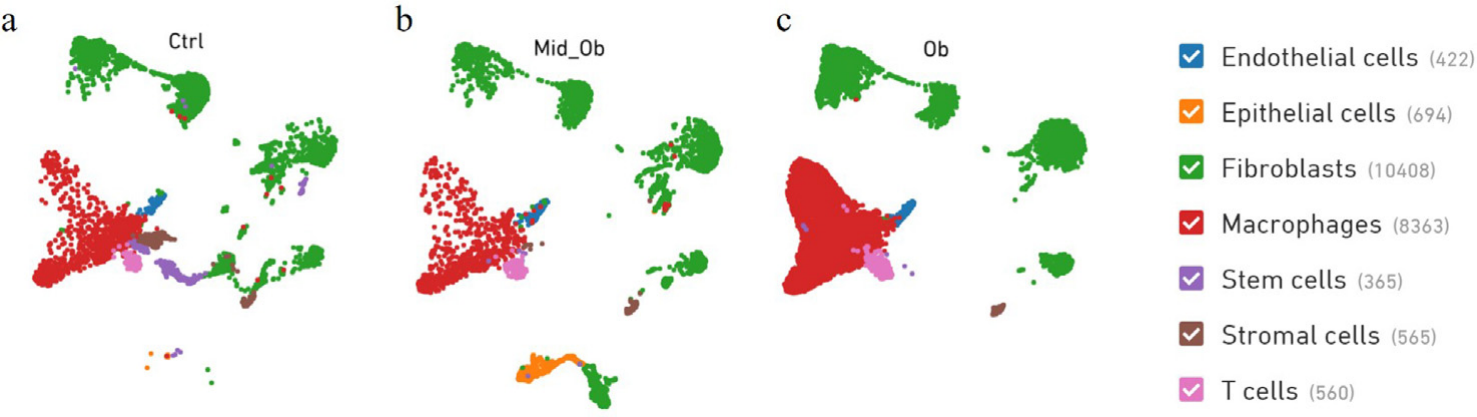
Adipose tissue was identified as seven cell types. Dataset GSM7502687 adipose tissue b. Dataset GSM7502688 adipose tissue c. Dataset GSM7502689 adipose tissue.

The single Cell sequencing process is as follows: (1) Raw data quality assessment (2) Quantitative quality control of Cell Ranger gene: Cell Ranger, the official software of 10x genomics, was used for sample quality control, and STAR [13] software was integrated in it. Reads were compared to the reference genome to obtain quality control results such as the number of high-quality cells, number of genes and genome comparison rate in the original data. Thus the quality of each sample is evaluated. (3) Quantitative post-quality control: Based on the preliminary quality control of Cell Ranger, further quality control of experimental data was carried out, and the data of multicellular, double-cell or uncombined upper cell were eliminated for downstream analysis. The quality control standards in this study are: Cells with more than 200 retained genes, more than 1000 UMI, more than 0.7 log10GenesPerUMI, less than 5% mitochondrial UMI, and less than 5% red blood cell genes were treated as high-quality cells and then double-cell removal was performed using DoubletFinder software [14]. Perform downstream analysis. (4) Standardized treatment of gene expression. Cell heterogeneity analysis: dimensionality reduction clustering, Marker gene identification, cell type identification, cell subsets and other downstream personalized analysis. ⑥ Gene expression analysis: differential gene analysis, differential gene enrichment analysis and other downstream personalized analysis. Fig 2 shows the main flow diagram of data acquisition. Adipose tissue was divided into 18 cell subsets by the reduced-dimension cluster analysis, different colors in the diagram represent different subpopulations of cells (Fig. 3). Adipose tissue has been identified as seven cell types, with different colors representing different cell types (Fig. 4), from the figure, we can see that fibroblasts and macrophages account for a large proportion, so we speculate that they play a key role in the development of adipose tissue obesity. The dimensionality reduction clustering diagram of GSM7502687 is shown in Figs. 3a and 4a. The dimensionality reduction clustering diagram of GSM7502688 is shown in Figs. 3b and 4b. The dimensionality reduction clustering diagram of GSM7502689 is shown in Figs. 3c and 4c. Table 4 is statistical table of differentially expressed genes.String_protein-protein-interaction *(p<0.05, FC>1.5*) was shown in Fig. 6. Gene Ontology Classification was shown in Fig. 7. KEGG Pathway Classification was shown in Fig. 8.

**Table 4:** Statistical table of differentially expressed genes.

Case	Control	Up_diff	Down_diff	Total_diff (*p<0.05, FC>1.5*)
Mid_Ob	Ctrl	317	390	707
Ob	Mid_Ob	373	449	822
Ob	Mid_Ob	467	679	1146

**Fig. 5:**
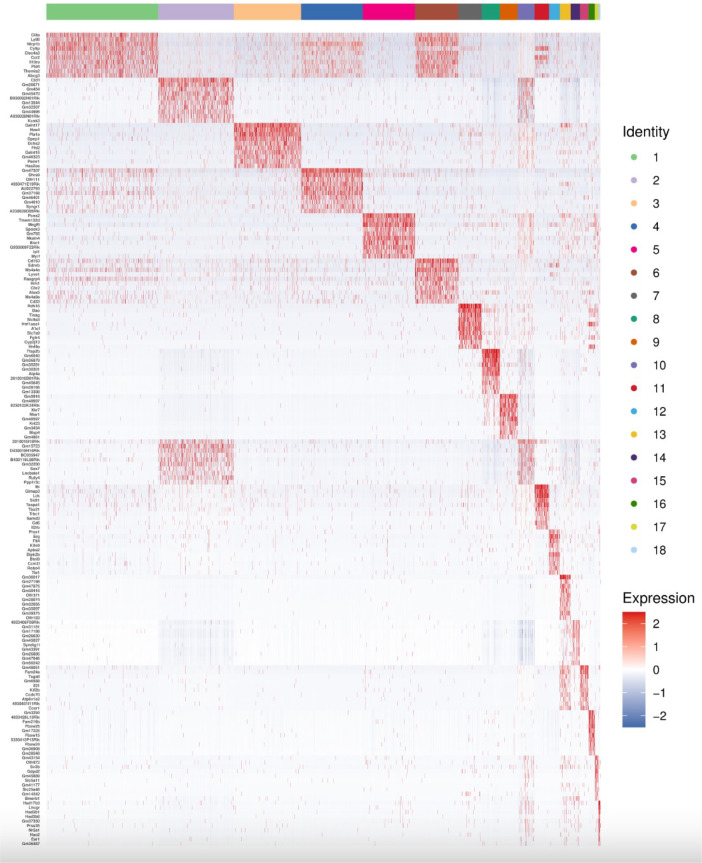
Heat map of Top10 Marker gene expression in each cell subgroup.

The horizontal coordinate is cell population, and the vertical coordinate is Marker gene. In the figure, red indicates high expression and blue indicates low expression (Fig. 5).

**Fig. 6:**
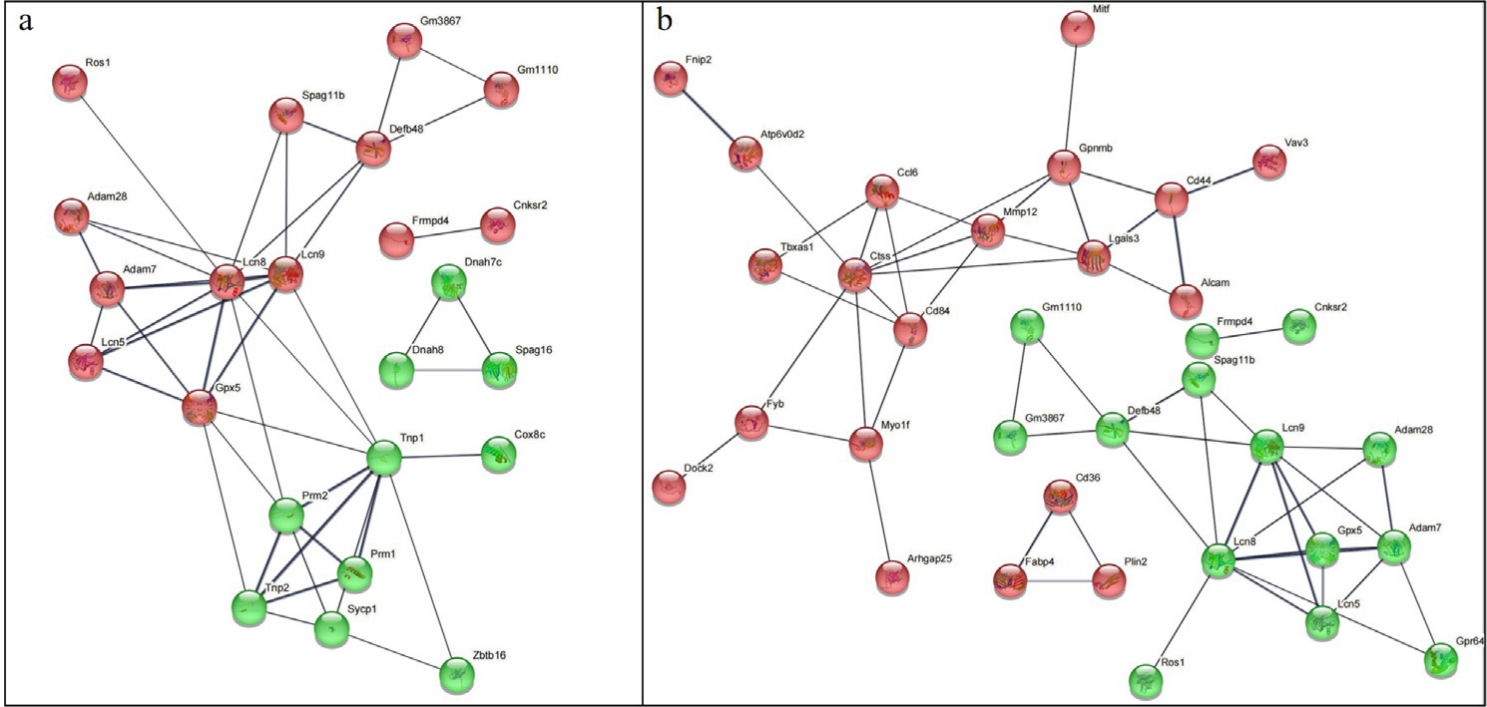
String_protein-protein-interaction (*p<0.05, FC>1.5*). a. sampleid_Mid_Ob-vs-Ctrl-diff string_protein-protein-interaction. b. sampleid_Ob-vs-Mid_Ob-diff string_protein-protein-interaction.

**Fig. 7:**
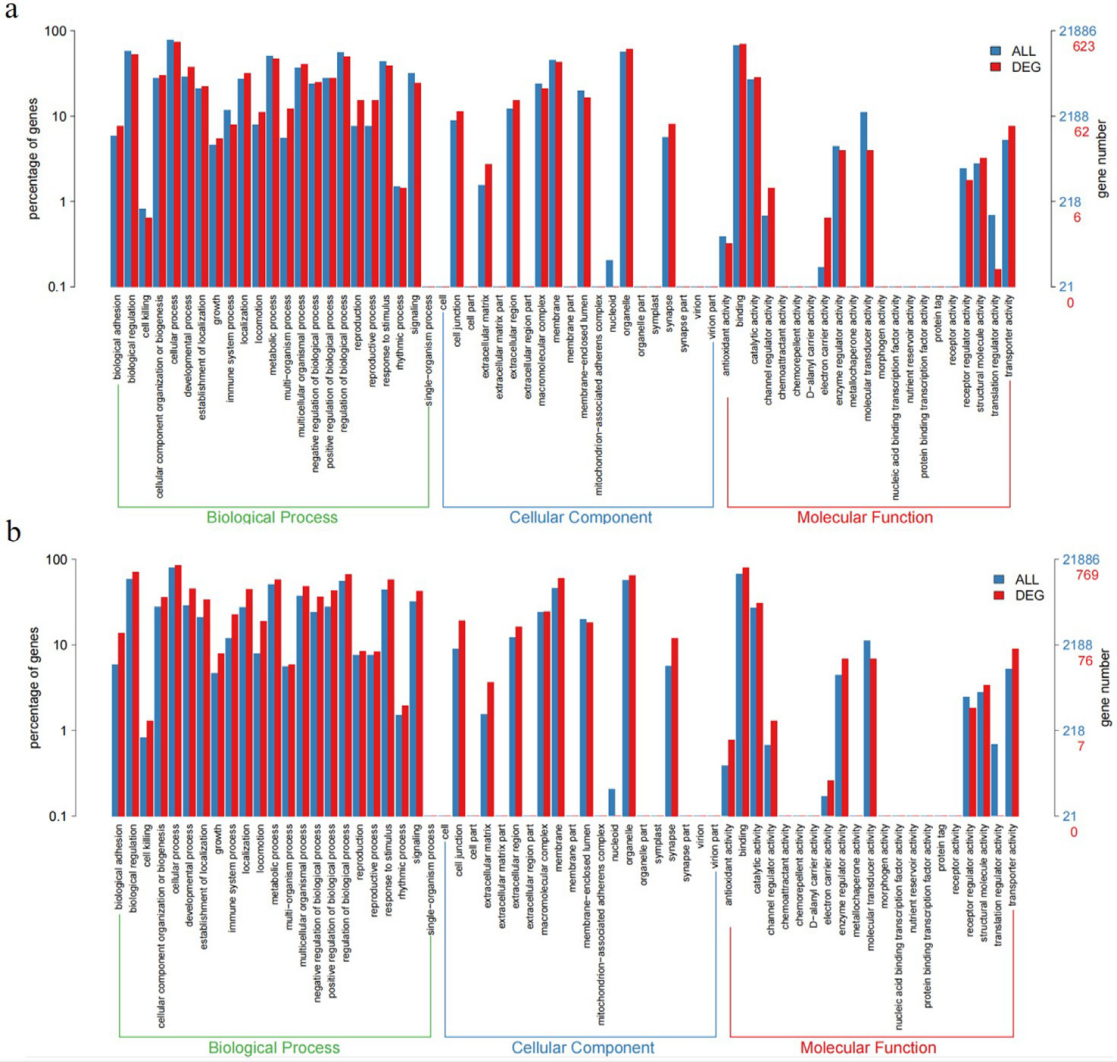
Gene Ontology Classification. a. Gene Ontology Classification (sampleid_Mid_Ob−vs−Ctrl). b. Gene Ontology Classification (sampleid_Ob−vs−Mid_Ob).

**Fig. 8:**
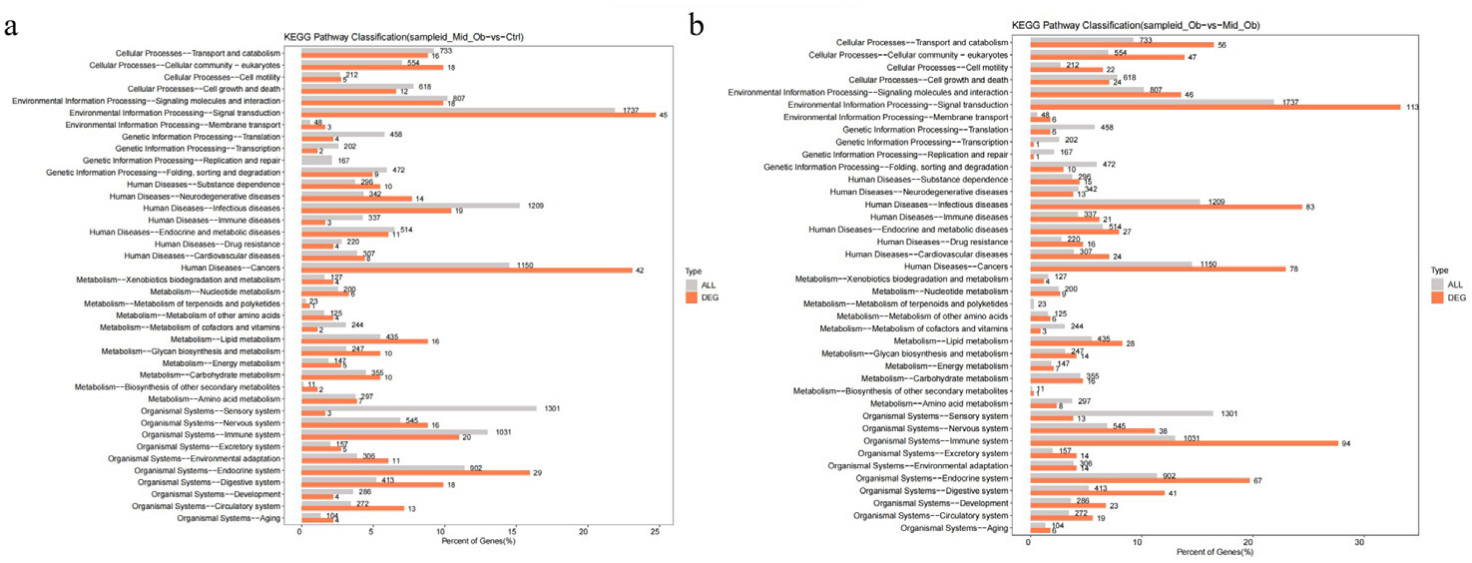
KEGG Pathway Classification. a. KEGG Pathway Classification (sampleid_Mid_Ob−vs−Ctrl). b. KEGG Pathway Classification (sampleid_Ob−vs−Mid_Ob).

## Limitations

Not applicable.

## Ethics Statement

The animals we selected were male C57BL/6J mice, All experiments were conducted in accordance with the National Institutes of Health Guidelines for the Care and Use of Experimental Animals (NIH Publication No. 8023, revised 1978). Approval for this study was provided by the Shandong Sport University Animal Ethics Committee (China).

## CRediT authorship contribution statement

**Zhimin Lu:** Conceptualization, Writing – original draft, Methodology, Software. **Ling Ding:** Data curation, Visualization, Investigation. **Xuewen Tian:** Supervision, Validation. **Qinglu Wang:** Writing – review & editing.

## Data Availability

Single cell sequencing reveals changes in the adipose tissue of the epididymis during the development of obesity in mice (Original data) (GEO).
